# Unveiling the Photodegradation Mechanism of Monochlorinated Naphthalenes under UV-C Irradiation: Affecting Factors Analysis, the Roles of Hydroxyl Radicals, and DFT Calculation

**DOI:** 10.3390/molecules29194535

**Published:** 2024-09-24

**Authors:** Yingtan Yu, Mengdi Liu, Shimeng Wang, Chaoxing Zhang, Xue Zhang, Li Liu, Shuang Xue

**Affiliations:** School of Environment, Liaoning University, Shenyang 110036, China; yuyingtan@lnu.edu.cn (Y.Y.); lmd99909@163.com (M.L.); wangshimeng0102@163.com (S.W.); 13842068408@163.com (C.Z.); zhangxuexiaonini@163.com (X.Z.)

**Keywords:** PCNs, organic pollutants, photodegradation, hydroxyl radicals, DFT

## Abstract

Polychlorinated naphthalenes (PCNs) are a new type of persistent organic pollutant (POP) characterized by persistence, bioaccumulation, dioxin-like toxicity, and long-range atmospheric transport. Focusing on one type of PCN, monochlorinated naphthalenes (CN-1, CN-2), this study aimed to examine their photodegradation in the environment. In this work, CN-1 and CN-2 were employed as the model pollutants to investigate their photodegradation process under UV-C irradiation. Factors like the pH, initial concentrations of CN-1, and inorganic anions were investigated. Next, the roles of hydroxyl radicals (^•^OH), superoxide anion radicals (O_2_^•−^), and singlet oxygen (^1^O_2_) in the photodegradation process were discussed and proposed via theory computation. The results show that the photodegradation of CN-1 and CN-2 follows pseudo-first-order kinetics. Acidic conditions promote the photodegradation of CN-1, while the effects of pH on the photodegradation of CN-2 are not remarkable. Cl^−^, NO_3_^−^, and SO_3_^2−^ accelerate the photodegradation of CN-1, whereas the effect of SO_4_^2−^ and CO_3_^2−^ is not significant. Additionally, the contributions of ^•^OH and O_2_^•−^ to the photodegradation of CN-1 are 20.47% and 38.80%, while, for CN-2, the contribution is 16.40% and 16.80%, respectively. Moreover, the contribution of ^1^O_2_ is 15.7%. Based on DFT calculations, C4 and C6 of the CN-1 benzene ring are prioritized attack sites for ^•^OH, while C2 and C9 of CN-2 are prioritized attack sites.

## 1. Introduction

Polychlorinated naphthalenes (PCNs) are a group of 75 theoretically possible chlorinated naphthalenes, containing from one to eight chlorine atoms [1]. PCNs have been listed as new persistent organic pollutants (POPs) due to their persistence, long-range transport, bioaccumulation, and potential for causing adverse effects in organisms [2]. Although the manufacture of PCNs has been prohibited in the majority of nations since the 1980s, these persistent organic pollutants continue to be detected within different environmental mediums such as the atmosphere, soil, water, sediments, and biota [3,4,5,6,7]. Nowadays, unintentional releases from thermal treatment such as the incineration of waste and metallurgical processes, from which PCNs are directly released into the atmosphere, are likely the main source of PCNs in the environment [1]. The homologue’s distributed profile is dominated in the atmosphere by less chlorinated naphthalenes, in which trichlorinated naphthalenes (tri-CNs) and tetrachlorinated naphthalenes (tetra-CNs) are the main homologues [8,9,10,11,12,13]. In atmospheric samples from Barcelona (Spain), monochlorinated naphthalenes (mono-CNs) and dichlorinated naphthalenes (di-CNs) are found whose concentrations are several magnitudes higher than other more highly chlorinated naphthalenes, which indicates that mono-CN is also a primary homologue in the atmosphere [14]. Atmospheric dispersion is considered an important pathway for the global distribution of semi-volatile and persistent environmental pollutants due to their long-range atmospheric transport ability [15]. PCNs have also been found in the polar regions, where mono-CNs dominate the total mass of Arctic air samples [16]. Moreover, the long-range transport potentials of several PCN homologs were assessed using QSAR models: high mobility (mono-CNs), relatively high mobility (di-CNs to tetra-CNs), relatively low mobility (pentachlorinated naphthalenes to hexachlorinated naphthalenes), and low mobility (heptachlorinated naphthalenes and octachloronaphthalene). This assessment indicates that mono-CNs could be transported globally in the stratosphere [2], where UV-C irradiation can be an important source for the conversion of mono-CNs.

There are rare reports involving the photodegradation of PCNs. Early studies on PCNs were mainly carried out in organic solvents or mixed organic–water solutions under simulated sunlight. They investigated less chlorinated naphthalenes (mono-CNs to tetra-CNs), octachloronaphthalene, and industrial mixtures of PCNs in methanol, ethanol, n-hexane, cyclohexane, benzene, and 4:1 acetonitrile–water solutions (*v*/*v*) under simulated sunlight. They found photoconversion processes of PCNs involving dechlorination and dimerization. Furthermore, methoxynaphthalene and its dimer were identified in methanol, 1-phenyl naphthalene was identified in benzene, and 1-naphthol in acetonitrile–water solution, and these were also found as products of photoconversions. These processes of products’ formation refer to the direct photolysis of the C-Cl bond, and radicals attacking naphthalene or benzene [17,18,19,20,21]. In recent years, the photodegradation of technical PCNs, and Halowax, under solar irradiation was investigated, and it was found that the most highly chlorinated octachloronaphthalene is much more stable than PCNs with a low molecular mass [22]. Moreover, the photodegradation of PCNs including CN-1 in various organic solvents under simulated sunlight by using a 400 W high-pressure mercury lamp as a light source has been reported, and it was found that the photoconversion process of PCNs went through two stages of dechlorination and oxidative ring opening [23]. Also, the photodegradation of 1-chlorinated naphthalene (CN-1), 2-chlorinated naphthalene (CN-2), and 2,3-dichlorinated naphthalenes (CN-10) in water at λ > 280 nm was investigated and it was verifyied that singlet oxygen (^1^O_2_) and hydroxyl radicals (^•^OH) were produced during photodegradation and positively affected the photodegradation process. In addition, possible photodegradation pathways of CN-1, CN-2, and CN-10 in water were proposed [24,25]. However, these reports mainly focus on the photoconversion of PCNs under simulated sunlight, while the mechanisms and processes of PCNs’ photodegradation in water under UV-C irradiation are still unclear.

In this work, the photodegradation of CN-1 and CN-2 under UV-C irradiation was investigated. The roles of ^•^OH and ^1^O_2_ in the photodegradation process were identified via radical quenching experiments and the second-order rate constants of CN-1/CN-2 with ^•^OH were measured using a competition method. The effects of pH and common inorganic anions (Cl^−^, CO_3_^2−^, SO_4_^2−^, NO_3_^−^, SO_3_^2−^) on the photodegradation of CN-1 and CN-2 were also considered. The primary photodegradation pathway was proposed using frontier electron density, transition-state theory, and intrinsic reaction coordinates calculations. The result of this study will help to further reveal the mechanism of the photodegradation of PCNs under UV-C irradiation.

## 2. Results and Discussion

### 2.1. Comparison of Dark and Light Reactions of CN-1 and CN-2

**Figure 1:**
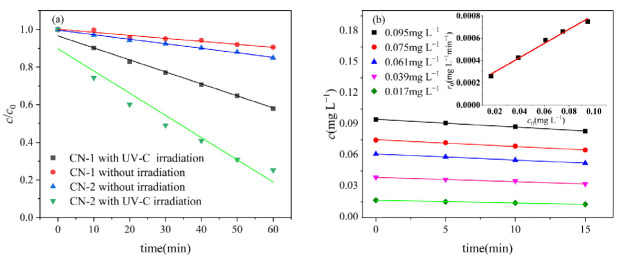
(**a**) Comparison of light and dark reactions between CN-1 and CN-2; (**b**) effect of initial concentration of CN-1 on photodegradation rate. Conditions: [CN-1]_0_ = 0.615 μmol L^−1^, [CN-2]_0_ = 0.615 μmol L^−1^, 20 °C.

Figure 1a illustrates the contrast between the light and dark reactions of CN-1. The UV-Vis absorption spectra of CN-1 and CN-2 are presented in Figure S2. In the dark controls, approximately a 10% loss of CN-1 was observed after 60 min due to the volatility of CN-1, while the volatile loss of CN-2 was slightly higher than that of CN-1 due to the low water solubility of CN-2. The photodegradation efficiency of CN-1 after 60 min of irradiation reached 42% under the medium-pressure mercury lamp. The absorption of 0.1 mg L^−1^ of CN-2 is higher than that of CN-1 at the wavelength band of 224 nm, as shown in Figure S2. As a result, the photodegradation reaction is strong, leading to about the 75% photodegradation of CN-2 in 60 min. This photodegrading rate and efficiency are similar compared to other chlorinated aromatic pollutants, such as the observed reaction rate constant (*k*_obs_) of 2,4-dichlorophenol in UV-C irradiation, which was 0.076 ± 0.004 min^−1^ [26], and that of 1,4-dichlorobenzene which was degraded to 56% in UV-C irradiation [27].

The kinetics of CN–1 photodegradation at different initial concentrations indicated that the photodegradation of CN-1 follows pseudo-first-order kinetics with a kinetic constant of 0.011 min^−1^, as shown in Figure 1b.

### 2.2. The pH Effect on the Photodegradation of CN-1 and CN-2

**Figure 2:**
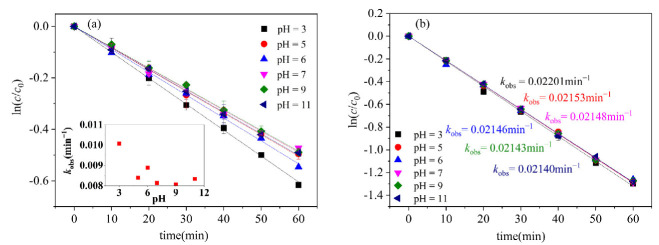
Effect of different pH on photodegradation of (**a**) CN-1, (**b**) CN-2. Conditions: [CN-1]_0_ = 0.615 μmol L^−1^, [CN-2]_0_ = 0.615 μmol L^−1^, 20 °C.

The pH value of a solution has varying effects on different systems and, at the same time, the presence of both acid and alkali also exhibits significant differences in the degradation of various substances [28,29]. Research has demonstrated that the pH value has a significant impact on the photodegradation of organic pollutants [30,31]. The effects of the pH were investigated by adjusting the initial pH with perchloric acid (HClO_4_) and sodium hydroxide (NaOH), shown in Figure 2. The findings indicate that the influence of pH on the photodegradation of CN-2 is not significant, which may be due to the fast reaction rate of CN-2; with pH 3, CN-1 has the fastest photodegradation rate, and the CN-1 photodegradation conversion was promoted under acidic conditions. Nevertheless, alkaline conditions had little effect on its photodegradation. It is reported that the aqueous solution could generate hydrating electrons under irradiation, and that these electrons react with O_2_ to produce superoxide anion radicals (O_2_^•−^) (Equation (1)) [25,32]. Usually at low pH levels, the photosensitization of O_2_^•−^ to produce hydrogen peroxide (H_2_O_2_) is promoted due to a higher H^+^ abundance (Equation (2)) [33], and, subsequently, H_2_O_2_ photolysis produces more ^•^OH (Equation (3)) [34]. The ^•^OH is highly oxidizing and reacts quickly with the target pollutants. Under alkaline conditions, the self-scavenging effect can produce H_2_O_2_ (Equation (4)), which may reduce the removal efficiency [35]. Therefore, under acidic conditions, ^•^OH can promote the photodegradation and transformation of CN-1. We reached a similar conclusion in regards to the photodegradation of polycyclic aromatic hydrocarbons, with ^•^OH exhibiting a higher oxidation potential under low pH conditions, and we noted that, under acidic conditions, the degradation rate of phenanthrene (PHE) accelerates [33].

(1) $$e_{aq}^{-}+O_{2}\to O_{2}^{•-}$$

(2) $$O_{2}^{•-}+2H^{+}\to H_{2}O_{2}+O_{2}$$

(3) H2O2+hv(λ<360 nm)→2OH•

(4) OH•+OH•→H2O2

### 2.3. Role of ^•^OH, O_2_^•−^ and ^1^O_2_

**Figure 3:**
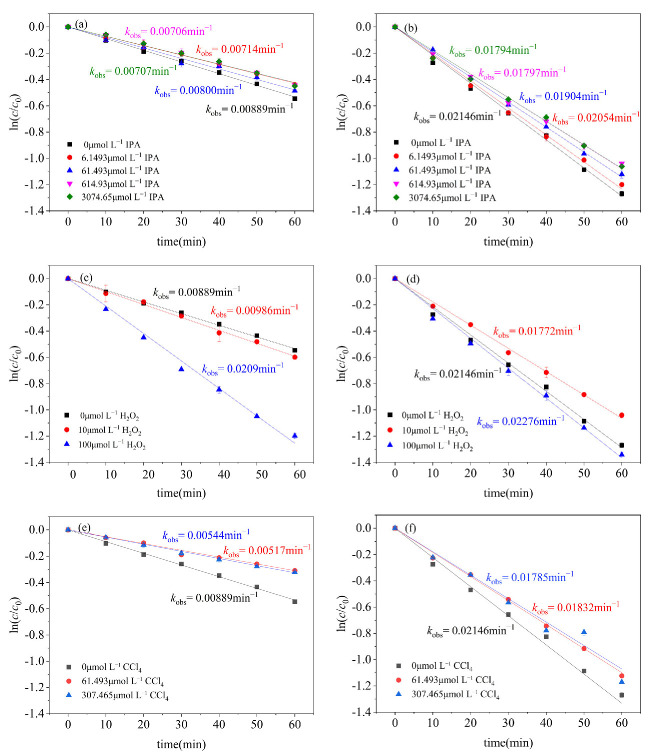
Effects of IPA on photodegradation of (**a**) CN-1, (**b**) CN-2; effects of H_2_O_2_ on photodegradation reaction of (**c**) CN-1, (**d**) CN-2; effects of CCl_4_ on photodegradation of (**e**) CN-1, (**f**) CN-2; conditions: [CN-1]_0_ = 0.615 μmol L^−1^, [CN-2]_0_ = 0.615 μmol L^−1^, 20 °C.

^•^OH is recognized as a highly oxidizing free radical that can participate in the non-selective degradation of many organic compounds at a very high reaction rate [36,37]. ^•^OH was quenched by adding isopropanol (IPA) as a scavenger during the photodegradation of CN-1 and CN-2 [38]. IPA has strong reactivity towards ^•^OH, with a second-order reaction rate of *k* _(IPA, •OH)_ = 1.9 × 10^9^ L mol^−1^s^−1^ [39,40]. It can be seen from Figure 3 that, with an increase in IPA concentration, the rate of the photodegradation of CN-1 and CN-2 decreased gradually. Also, the inhibition effect of IPA on the photodegradation of CN-1 and CN-2 remained stable and almost unchanged when the concentration of IPA was increased to 3074.65 μmol L^−1^. As a result, the contribution of ^•^OH to CN-1 degradation was 20.47%, and that for CN-2 was 16.40%, respectively. However, the influence of IPA on the photodegradation of CN-1 and CN-2 was not obvious because of the lower amount of ^•^OH produced during the photodegradation of CN-1 and CN-2.

To further determine the role of ^•^OH in the photodegradation conversion system, H_2_O_2_ was added to the CN-1 and CN-2 photoreaction systems. H_2_O_2_ is a very simple and stable molecule that serves as a reservoir for ^•^OH, which can be produced by the photolysis of H_2_O_2_, as shown in Equation (4) [41]. When the H_2_O_2_ concentration was 10 μmol L^−1^ and 100 μmol L^−1^, the photodegradation rate of CN-1 increased by 10.91% and 54.7%, respectively, as presented in Figure 3c. With an increase in the H_2_O_2_ concentration, the photolysis produced more ^•^OH, which greatly promoted the degradation of CN-1. However, the photodegradation of CN-2 was inhibited by the addition of 10 μmol L^−1^ H_2_O_2_, exhibitng an inhibition rate of 17.42% (Figure 3d), because H_2_O_2_ has strong absorption in UV-C and can compete with CN-2 for UV-C irradiation [42]. The amount of H_2_O_2_ of 100 μmol L^−1^ promoted the photodegradation of CN-2 with an acceleration rate of 6.06%, which was determined by the amount of ^•^OH produced by H_2_O_2_ photolysis.

As far as we know, there are no second-order rate constants of CN-1 and CN-2 with ^•^OH in the literature. Therefore, we performed a competitive experiment to determine the rate constants. Rhodamine B (RhB) was selected as the competent, and the values of *k*_(CN-1, •OH)_ and *k* _(CN-2, •OH)_ were calculated as 1.15 × 10^10^ L mol^−1^ s^−1^ and 1.9 × 10^10^ L mol^−1^s^−1^, respectively (Figure S3).

O_2_^•−^ has a good oxidizing ability for organic compounds [43] and, with UV irradiation, monochlorinated naphthalenes could elect electrons trapped by oxygen in an aerated medium to generate O_2_^•−^, which is similar to the photosensitive reaction of naphthalene [44]. Carbon tetrachloride (CCl_4_), an effective electron acceptor, can hinder the acceptance of electrons by dissolved oxygen to form O_2_^•−^. Therefore, CCl_4_ has been used as a scavenger for O_2_^•−^ in our experiments. From Figure 3e,f, it can be seen that, as the concentration of CCl_4_ increases, the *k*_obs_ gradually decrease. When the concentration increases to 307.465 μmol L^−1^, the *k*_obs_ gradually stabilizes; the contribution of O_2_^•−^ to CN-1 was 38.80%, and that on CN-2 was 16.80%, respectively.

**Figure 4:**
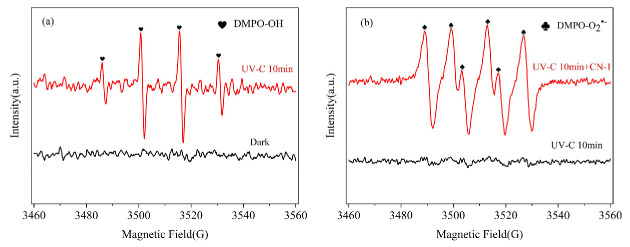
EPR spectra for different systems with (**a**) DMPO-^•^OH; (**b**) DMPO-O_2_^•−^.

An EPR analysis with DMPO as spin-trapping agents was further performed to recognize the ^•^OH and O_2_^•−^ produced in this system. The DMPO-^•^OH (1:2:2:1 with αN = 14.8 G, αH = 14.6 G) [45] values were detected and are shown in Figure 4a. Strong signals were found for DMPO-O_2_^•−^ adducts (αN = 14.2 G, αH = 11.2 G, and αH = 1.3 G) [46] in the EPR spectrum (Figure 4b), suggesting that O_2_^•−^ could stem from the addition of CN-1, while, without CN-1, the O_2_^•−^ signals were not found. This confirms what was mentioned earlier with regard UV irradiation: monochlorinated naphthalenes will undergo photosensitization to produce O_2_^•−^ (Figure S4).

^1^O_2_ is a highly reactive form of molecular oxygen and is recognized as a strong oxidizing agent. To investigate its effects, ^1^O_2_ was quenched by adding furfuryl alcohol (FAA) and sodium azide (NaN_3_) as scavengers during the photodegradation of CN-1 (*k*_(FFA_-_1O2)_ = 1.2 × 10^8^ M^−1^s^− 1^, *k*_(NaN3_-_1O2)_ = 2 × 10^9^ M^−1^s^−1^) [47,48]. When the concentration of added FFA was from 6.15 to 307.465 μmol L^−1^, it had little effect on the reaction, with only 10% being inhibited (Figure S5a). Furthermore, when increasing the concentration of NaN_3_, the photodegradation rate of CN-1 decreases. The inhibition of the CN-1 photodegradation by NaN_3_ reached its limit when the concentration of NaN_3_ increased to 1229.86 μmol L^−1^ (Figure S5b), where the inhibition ratio was 15.7% for its addition to ^1^O_2_.

### 2.4. The Influence of Typical Inorganic Ions on CN-1 in Natural Water

Natural water contains common inorganic ions such as Cl^−^, CO_3_^2−^, SO_4_^2−^, and NO_3_^−^ [49], some of which are photoactive and can produce reactive species to carry out the photodegradation of pollutants [50].

**Figure 5:**
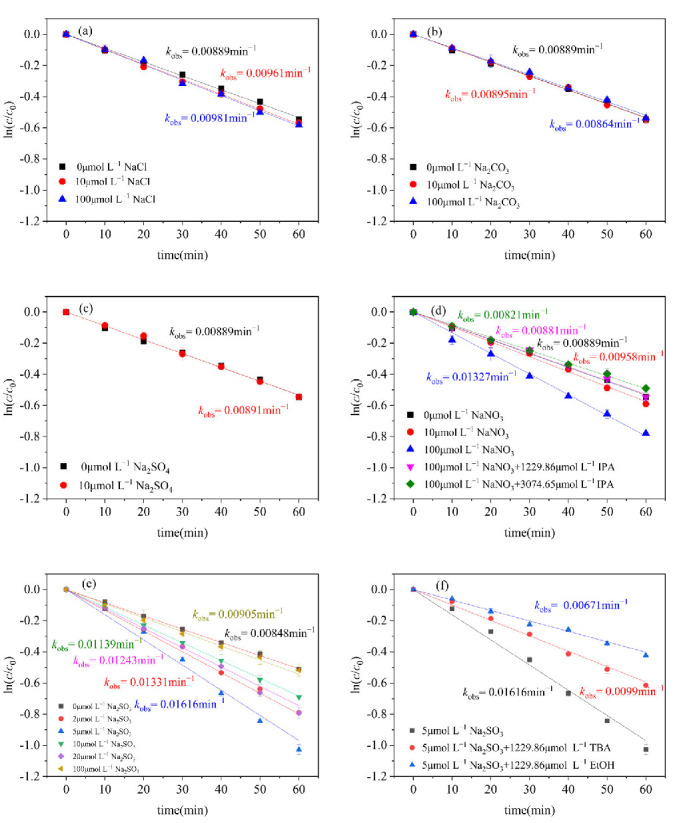
Effect of different substances on the photodegradation of CN–. (**a**) NaCl; (**b**) Na_2_CO_3_; (**c**) NaSO_4_; (**d**) NaNO_3_; (**e**) Na_2_SO_3_; (**f**) effects of TBA and EtOH on the photodegradation of CN-1 with Na_2_SO_3_. Conditions: [CN-1]_0_ = 0.615 μmol L^−1^, 20 °C.

The impact of Cl^−^ on the photodegradation process was examined, as illustrated in Figure 5a, with both 10 and 100 μmol L^−1^ of Cl^−^ accelerating the photodegradation of CN-1. This could be caused by the generation of chlorine radicals (Cl^•^) which accelerate the degradation of CN-1. Specifically, Cl^−^ can be converted into the free radical ^•^HOCl^−^, which then forms Cl^•^ radicals that promote the degradation of CN-1 (Equations (5) and (6)) [51]. Some studies show that Cl^•^ radicals formed from Cl^−^ can accelerate the degradation of PHE. Additionally, it was found that the photochemical degradation rate of PHE rises with the elevation of Cl^−^ levels [51,52].

(5) OH•+Cl−→HOCl-•

(6) HOCl-•+H+→Cl•+H2O

When adding different concentrations of Na_2_CO_3_ or NaSO_4_, the rate constant of CN-1 remains unchanged, indicating that the effect of CO_3_^2−^ and SO_4_^2−^ on the photocatalytic conversion of CN-1 is not significant (Figure 5b,c).

NO_3_^−^, as a photosensitizer, has a significant effect on the photodegradation of CN-1, as shown in Figure 5d. When elevating the NO_3_^−^ concentration from 10 to 100 μmol L^−1^, the *k*_obs_ of degradation was strengthened from 0.00958 min^−1^ to 0.01327 min^−1^. This intensification can be attributed to the amount of ^•^OH generated through the photolysis of NO_3_^−^, which promotes the degradation of CN-1 (Equation (7)) [53]. NO_3_^−^ can significantly promote the light conversion of CN-1, as demonstrated in a previous study [23]. Some reports have also confirmed that the production of ^•^OH under sunlight has a positive correlation with the nitrate concentration [33,54]. To further attest the effects of ^•^OH produced during the NO_3_^−^ photolysis process, IPA was used as the scavenger, the results of which are shown in Figure 5d. By adding 1229.86 μmol L^−1^ and 3074.65 μmol L^−1^ of IPA, the promoting effects of NO_3_^−^ were almost completely inhibited, which further proves that ^•^OH produced by photosensitive NO_3_^−^ promotes the photodegradation of CN-1.

(7) NO3−+H++hv→NO2•+OH•

Additionally, SO_3_^2−^ can be present in atmospheric droplets during acid rain processes [55,56] and, thus, could contribute to the photodegradation of CN-1. Because of this, Na_2_SO_3_ was added to investigate the effect of SO_3_^2−^ on the photodegradation of CN-1. The result (Figure 5e) shows that, when the presence of Na_2_SO_3_ increases from 0 μmol L^−1^ to 5 μmol L^−1^, the *k*_obs_ gradually increases, with the CN-1 degradation efficiency able to rise to 90.56% at its best performance, which is attributed to the increased reactivity of the sulfur oxygen species. SO_3_^2−^ activated by UV light will produce SO_3_^•−^ and hydrated electrons (e_aq_^−^) (Equation (8)), and e_aq_^−^ can react quickly with O_2_ in water, forming O_2_^•−^ (Equation (9)) [57]. Ultimately, the e_aq_^−^ will transform into H_2_O_2_ (Equations (10)-(12)) [58]. When SO_3_^•−^ reacts with O_2_ to generate SO_5_^•−^, it triggers a series of chain reactions in which SO_4_^•−^ and ^•^OH as the oxidative radicals are formed therein and play a promoting role in the photodegradation process (Equations (13)–(16)) [58]. When further elevating the SO_3_^2−^ concentration from 5 to 100 μmol L^−1^, the efficiency is lower, which is due to excess SO_3_^2−^ reducing the concentrations of SO_4_^•−^ through Equations (17)–(19) [59].

(8) $${SO}_{3}^{2-}+hv\to {SO}_{3}^{•-}+e_{aq}^{-}$$

(9) $$e_{aq}^{-}+O_{2}\to O_{2}^{•-}$$

(10) $$O_{2}^{•-}+H^{+}\to {HO}_{2}$$

(11) $$O_{2}^{•-}+{HO}_{2}\to {HO}_{2}^{•-}+O_{2}$$

(12) $${HO}_{2}^{•-}+H^{+}\to H_{2}O_{2}$$

(13) $${SO}_{3}^{•-}+O_{2}\to {SO}_{5}^{•-}$$

(14) $${SO}_{5}^{•-}+{HSO}_{3}^{-}\to H^{+}+{SO}_{4}^{2-}+{SO}_{4}^{•-}$$

(15) SO4•−+OH−→SO42−+OH•

(16) SO4•−+H2O→HSO4−+OH•

(17) $${SO}_{5}^{•-}+{HSO}_{3}^{-}\to {HSO}_{5}^{-}+{SO}_{3}^{•-}$$

(18) $${SO}_{4}^{•-}+{HSO}_{3}^{-}\to {SO}_{4}^{2-}+H^{+}+{SO}_{3}^{•-}$$

(19) $${HSO}_{5}^{-}+{HSO}_{3}^{-}\to {2SO}_{4}^{2-}+H_{2}O$$

Ethanol (EtOH) can react rapidly with SO_4_^•−^ (*k* = 1.6 × 10^7^–7.7 × 10^7^ M^−1^s^−1^) and ^•^OH (*k* = 1.2 × 10^9^ − 2.8 × 10^9^ M^−1^s^−1^), while tert-butanol (TBA) mainly reacts with ^•^OH, with a rate constant (*k* = 3.8 × 10^8^ − 7.6 × 10^8^ M^−1^s^−1^) that is significantly higher than that of the reaction with SO_4_^•−^ (*k* = 4 × 10^5^ − 9.1 × 10^5^ M^−1^s^−1^) [60]. Hence, to ascertain the roles of SO_4_^•−^ and ^•^OH, EtOH and TBA were used as radical scavengers in the photodegradation of CN–1 during the photolysis process of SO_3_^2−^. As a result, it was found that TBA selectively captures only ^•^OH, while EtOH captures both SO_4_^•−^and ^•^OH. As shown in Figure S6a, as the concentration of TBA in the system increases from 61.493 to 307.465 μmol L^−1^, the *k*_obs_ gradually decreases from 0.01616 to 0.01036 min^−1^. When the concentration of TBA increases from 307.465 μmol L^−1^ to 1229.86 μmol L^−1^, there is no significant change in the *k*_obs_, indicating that sufficient TBA completely inhibits ^•^OH. After increasing the concentration of TBA from 1229.86 to 4949.14 μmol L^−1^, the *k_obs_* slightly changes, indicating that excessive TBA inhibits the effects of SO_4_^•−^. As shown in Figure S6b, it is evident that, as the concentration of EtOH in the system ascends, the *k*_obs_ descends remarkably, down to 0.00671 min^−1^ with 1299.86 μmol L^−1^ EtOH. This is attributed to the fact that ^•^OH and SO_4_^•−^ are completely inhibited by EtOH during the photolysis of SO_3_^2−^. As shown in Figure 5f, the contributions of SO_4_^•−^ and ^•^OH can be further calculated to show that the contribution of ^•^OH to the photodegradation of CN-1 was 38.74%, while the contribution of SO_4_^•−^ to the photodegradation of CN-1 was 19.74%.

### 2.5. Photodgrdation of 1-Naphthol and Naphthalene

It is known from previous studies that the two products of CN-1 photodegradation are 1-naphthol and naphthalene [24,25]. However, the photodegradation products of CN-1 were not detected by gas chromatography-mass spectrometry (GC-MS) in this experiment. The photodegradation experiments for naphthalene and 1-naphthol were carried out for 0, 10, 20, 30, 40, 50, and 60 min, with the 1.5 mL photodegradation samples being determined by liquid chromatography. The *k*_obs_ of the photodegradation of naphthalene is 0.01667 min^−1,^ and that of 1-naphthol is 0.06646 min^−1^ (Figure S7). Given identical circumstances, the degradation rates of naphthalene and 1-naphthol are much faster than that of CN-1. When processing the photodegradation of CN-1, the productions of naphthalene and 1-naphthol were quickly converted into smaller substances, so the productions of naphthalene and 1-naphthol were not detected in gas chromatography-mass spectrometry.

### 2.6. Density Functional Theory (DFT)

DFT has a wide range of applications and is an invaluable tool in scientific research, providing information on the structure and properties of compounds while further exploring their reaction behavior and intrinsic mechanisms [61,62]. DFT calculations were used to further determine the relevant reaction mechanisms of CN-1 and CN-2 and to explore the products and degradation pathways.

**Figure 6:**
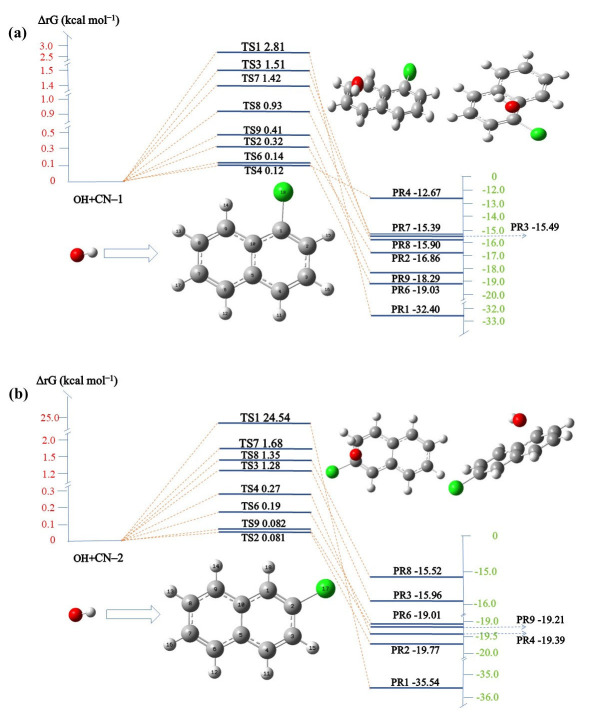
Calculated reaction barriers (ΔrG) for the ^•^OH; (**a**) CN-1; (**b**) CN-2.

According to the frontier electron density (FED) the chemistry of the molecule is closely related between the highest occupied molecular orbital (HOMO) and lowest unoccupied molecular orbital (LUMO), with electrophilic reactions being most likely to occur at 2FED^2^_HOMO_ and atoms with larger charge distributions, while free radicals are prone to attacking the higher positions of FED^2^_HOMO_ + FED^2^_LUMO_ [63,64]. As can be seen from Table 1, the highest value of FED^2^_HOMO_ + FED^2^_LUMO_ among the unsaturated carbons occurs at C4 (0.26946), followed by the C1 (0.25793), C9 (0.23643), and C6 (0.2356) atoms. This result suggests that these carbons are susceptible to free radical attack. In CN-2, the FED^2^_HOMO_ + FED^2^_LUMO_ values of the C1, C9, C4, and C6 sites are higher, as shown in Table 2, which indicates that the free radicals will preferentially attack the above sites during the reaction process. In CN-1, the values of 2FED^2^_HOMO_ at C4, C1, and C9 are the highest, which means that electrons at these positions are more vulnerable to electron extraction, while, in CN-2, the C1, C9, and C6 sites are more vulnerable to electron extraction [65]. The changes in energy of CN-1 and CN-2 during their degradation by ^•^OH were additionally assessed using TS theory, with the results depicted in Figure 6. CN-1 has eight reaction sites in the benzene ring, namely C1–C4 and C6–C9 [66]. The lower the reaction barriers (ΔrG), the easier it is to attack [67], and the energies of TS4 and TS6 are 0.12 kcal mol^−1^ and 0.14 kcal mol^−1^, which are very close in proximity. Therefore, the reaction between ^•^OH and CN-1 will prioritize attacking C4 and C6 connected to the benzene ring. In the reaction of ^•^OH with CN-2, the energies of TS2 and TS9 are 0.0081 kcal mol^−1^ and 0.0082 kcal mol^−1^, respectively. Therefore, C2 and C9 are attacked first, with the substitution reaction between ^•^OH and Cl occurring at the C2 site, while those at all other C sites are addition reactions. Research indicates that the ^•^OH addition route possesses a lower energy barrier and is more likely to take place compared to the H abstraction route [68], while ^•^OH with C-Cl prefers substitution in its reactions [69,70].

Based on the FED results and TS theory, it can be surmised that free radicals will show a propensity for attacking the benzene ring moieties of CN-1 and CN-2. This preferential attack induces the benzene rings to open. Nevertheless, the abundance of potential attack sites leads to an overly rapid and extensive fragmentation during the ring opening of CN-1 and CN-2, resulting in fragmentation patterns that are elusive to detection by techniques such as gas chromatography-mass spectrometry (GC-MS) [71]. Thus, it is crucial to comprehensively examine the reaction mechanisms of CN-1 and CN-2 with the ^•^OH group.

## 3. Materials and Methods

### 3.1. Reagents

In this study, CN-1 (98% purity), CN-2 (98% purity), 1-naphthol (98% purity), naphthalene (98% purity), IPA, NaN_3_, FFA, H_2_O_2_ (30%, *v*/*v*), HClO_4_, NaOH, NaCl, NaSO_4_, Na_2_CO_3_, NaNO_3_, Na_2_SO_3,_ EtOH, and ferrous sulfate heptahydrate were obtained from Sinopharm Chemical Reagent Co., Ltd. (Shanghai, China). The methanol and acetonitrile used are all chromatographically pure, being purchased from Thermo Fisher Scientific Inc. (Fair Lawn, NJ, USA). The RhB and TBA were purchased from Aladdin Biochemical Technology Co., Ltd. (Shanghai, China). All reagents with no specification are analytical grades. Ultrapure water (≥18.2 MΩ cm) was used throughout the entire experiment. The detailed usage information of the reagents is listed in the Supplementary Materials (Text S1).

### 3.2. Photodegradation Experiments

The photochemical degradation experiments were carried out in a 500 mL self-designed photodegradation device, which was previously used for the photodegradation of hexabromocyclododecane (HBCD) [42], as shown in Figure S1. A 14 W UV-C lamp immersed directly in the 500 mL reaction solution was used as the light source, with the part of the lamp above the liquid level wrapped with tin foil. Reactions were initiated by adding 0.615 μmol L^−1^ CN-1/CN-2 into solutions containing acetonitrile–water (1‰, *v*/*v*). Except when otherwise mentioned, experiments were performed at room temperature (20 ± 2 °C) under open atmospheric conditions. At specific time intervals, every 10 min, a 2 mL sample was then taken out and analyzed with HPLC. Each set of experiments was repeated at least in triplicate, with the mean values with error bars being calculated and reported.

### 3.3. Competitive Reaction

A competitive reaction was designed to determine the second-order rate constants of CN-1 and CN-2 with ^•^OH. RhB was employed as the competitive chemical to react with ^•^OH. The detailed experimental methods are described in our previous study [42].

### 3.4. Samples Analysis

High-performance liquid chromatography (HPLC) was utilized to analyze the content of CN-1 and CN-2 in the sample. The sample was separated with a reversed-phase C18 column (250 mm × 4.6 mm, SHIMADZU (China) Co., Ltd., Shanghai, PRC) at 25 °C column temperature. The mobile phase consisted of 90% methanol and 10% water, with a flow rate of 1 mL min^−1^ and detection at a wavelength of 225 nm. The UV absorption spectra of CN-1 and CN-2, as well as the concentration of Rhodamine B, were determined using an ultraviolet spectrophotometer (Varian Technology China Co., Ltd., Beijing, China).

### 3.5. Density Functional Theory (DFT) Calculation

Optimization of the CN-1 and CN-2 molecular structure was performed with density functional theory (DFT)/B3LYP/6-311G(2d, p) level via Gaussian 09 software [72]. After optimization, Multiwfn Version 3.8 software was employed to calculate the contribution of each atomic orbital for frontier electron density (FED) analyses (HOMO and LUMO) [73] and to predict the most reactive part for degradation of CN-1 and CN-2, and the results were visualized and analyzed with the help of the Gaussview 6.0 program.

Transition states (TSs) were searched by the pseudo-reaction coordinate method and further confirmed by intrinsic reaction coordinate (IRC) analysis. Vibrational frequency calculations were performed to ensure a true energy minimum, and IRC analyses were to confirm a first-order saddle point [70]. From IRC analysis, the initial guess structures of reactant complexes (REs) and product complexes (PRs) were also obtained, which were subsequently optimized with the same method and basis set of CN-1 and CN-2. The plausibility of the reaction and the prior reaction channel were determined by the reaction energy barriers (ΔrG, ΔrG = GTS-GRE) [74].

## 4. Conclusions

Monochlorinated naphthalene (CN-1, CN-2) can be photodegraded under UV-C irradiation, with this photodegradation following the pseudo-first-order kinetics. In acidic conditions, the photodegradation of CN-1 accelerates while, in alkaline conditions, the photodegradation is almost unaffected. The second-order rate constant between CN-1 and ^•^OH (*k* _(CN-1, •OH)_) was determined to be 1.15 × 10^10^ L mol^−1^s^−1^, while the *k* _(CN-2, •OH)_ was determined to be 1.9 × 10^10^ L mol^−1^s^−1^. The contributions of ^•^OH and O_2_^•−^ to CN-1 degradation were 20.47% and 38.80%, those for CN-2 were 16.40% and 16.80%, respectively, and the contribution to ^1^O_2_ was 15.7%. Additionally, Cl^−^, NO_3_^−^, and SO_3_^2−^ can enhance the photodegradation efficiency of CN-1 and CN-2, while the effects of SO_4_^2−^ and CO_3_^2−^ were not significant. Through DFT calculation, it can be determined that ^•^OH reacts the most favorably with the C4 and C6 sites in CN-1, whereas ^•^OH reacts the most favorably with the C2 and C9 sites in CN-2.

## Figures and Tables

**Table 1:** Results of frontier electron density for CN-1.

Sequence	Bond	Bond Orders	Charge Distribution	HOMO	LUMO	2FED^2^_HOMO_	FED^2^_HOMO_ + FED^2^_LUMO_
C1	C-C	2.0	0.036	0.13134	0.12659	0.26268	0.25793
C2	C-C	1.5	−0.060	0.08247	0.09041	0.16494	0.17288
C3	C-C	2.0	−0.063	0.01969	0.07948	0.03938	0.09917
C4	C-C	1.5	−0.093	0.14295	0.12651	0.2859	0.26946
C5	C-C	1.5	0.132	0.02539	0.03534	0.05078	0.06073
C6	C-C	2.0	−0.098	0.11724	0.11836	0.23448	0.2356
C7	C-C	1.5	−0.055	0.06314	0.08009	0.12628	0.14323
C8	C-C	2.0	−0.046	0.07201	0.08315	0.14402	0.15516
C9	C-C	1.5	−0.109	0.11869	0.11774	0.23738	0.23643
C10	C-C	1.5	0.071	0.02577	0.03584	0.05154	0.06161
H11	C-H	1.0	0.050	0.0118	0.01322	0.0236	0.02502
H12	C-H	1.0	0.049	0.00953	0.0121	0.01906	0.02163
H13	C-H	1.0	0.054	0.00579	0.00902	0.01158	0.01481
H14	C-H	1.0	0.082	0.0108	0.01305	0.0216	0.02385
H15	C-H	1.0	0.077	0.00583	0.01034	0.01166	0.01617
H16	C-H	1.0	0.055	0.00626	0.00853	0.01252	0.01479
H17	C-H	1.0	0.053	0.00491	0.0086	0.00982	0.01351
Cl18	C-Cl	1.0	−0.133	0.08638	0.03162	0.17276	0.118

**Table 2:** Results of frontier electron density for CN-2.

Sequence	Bond	Bond Orders	Charge Distribution	HOMO	LUMO	2FED^2^_HOMO_	FED^2^_HOMO_ + FED^2^_LUMO_
C1	C-C	2.0	−0.147	0.14654	0.12779	0.29308	0.27433
C2	C-C	1.5	0.091	0.09376	0.08161	0.18752	0.17537
C3	C-C	2.0	−0.098	0.04642	0.08777	0.09284	0.13419
C4	C-C	1.5	−0.106	0.1127	0.13552	0.2254	0.24822
C5	C-C	1.5	0.146	0.02653	0.03621	0.05306	0.06274
C6	C-C	2.0	−0.107	0.12828	0.11779	0.25656	0.24607
C7	C-C	1.5	−0.055	0.09626	0.07802	0.19252	0.17428
C8	C-C	2.0	−0.050	0.05834	0.08388	0.11668	0.14222
C9	C-C	1.5	−0.111	0.13813	0.11598	0.27626	0.25411
C10	C-C	1.5	0.146	0.03135	0.03532	0.0627	0.06667
H11	C-H	1.0	0.054	0.00928	0.01428	0.01856	0.02356
H12	C-H	1.0	0.049	0.01043	0.01206	0.02086	0.02249
H13	C-H	1.0	0.053	0.00425	0.00918	0.0085	0.01343
H14	C-H	1.0	0.050	0.01124	0.01180	0.02248	0.02304
H15	C-H	1.0	0.075	0.00352	0.00898	0.00704	0.0125
H16	C-H	1.0	0.052	0.00794	0.00835	0.01588	0.01629
Cl17	C-Cl	1.0	−0.113	0.06386	0.02146	0.12772	0.08532
H18	C-H	1.0	0.072	0.01115	0.01398	0.0223	0.02513

## Data Availability

Data is contained within the article. The materials used in the study are commercially available and can be purchased from the relevant firms.

## Supplementary Materials

The following supporting information can be downloaded at: https://www.mdpi.com/article/10.3390/molecules29194535/s1, Figure S1 Photodegradation device. Figure S2 UV-C lamp emission spectrum, UV absorption spectra of CN-1 and CN-2. Figure S3 Concentration variation in a competitive reaction (a) CN-1 and RhB; (b) CN-2 and RhB. Figure S4 Scheme for generating O_2_^•−^ by monochlorinated naphthalene under UV irradiation. Figure S5 Effects of ^1^O_2_ scavengers on photodegradation of CN-1 (a) FFA (b) NaN_3_. Figure S6 Effects of scavengers on the photodegradation of CN-1 with Na_2_SO_3_(a) TBA; (b) EtOH. Figure S7 photodegradation experiment (a) naphthalene (b) 1-naphthol. Text S1. Specific use of reagents.
