# Supplementary material for: Unveiling the Photodegradation Mechanism of Monochlorinated Naphthalenes under UV-C Irradiation: Affecting Factors Analysis, the Roles of Hydroxyl Radicals, and DFT Calculation
Source: Molecules. 2024 Sep 24;29(19):4535. doi: 10.3390/molecules29194535 (PMC11477601; doi:10.3390/molecules29194535)
Supplement: Supplementary file 1 [file molecules-29-04535-s001.zip › molecules-3149432-supplementary.pdf]

Supplementary Material

for

Unveiling the photodegradation mechanism of monochlorinated naphthalenes under UV–C irradiation: affecting factors analysis, the roles of hydroxyl radicals and DFT calculation

Lists of captions:

Figure S1 Photodegradation device 3

Figure S2 UV–C lamp emission spectrum, UV absorption spectra of CN–1 and CN–2 3

Figure S3 Concentration variation in a competitive reaction (**a**)CN–1 and RhB; (**b**)CN–2 and RhB ....5~~4~~

Figure S4 Scheme for generating $O_2^{\bullet -}$ by Monochlorinated naphthalene under UV irradiation ....6~~5~~

Figure S5 Effects of $^1O_2$ scavengers on photodegradation of CN–1 (**a**)FFA (**b**)NaN_3 ....8~~6~~

Figure S6 Effects of scavengers on the photodegradation of CN–1 with Na_2SO_3(**a**) TBA; (**b**) EtOH ....10~~7~~

Figure S7 photodegradation experiment (**a**) naphthalene (**b**) 1−naphthol...............12~~8~~

Text S1. Specific use of reagents ....13~~8~~

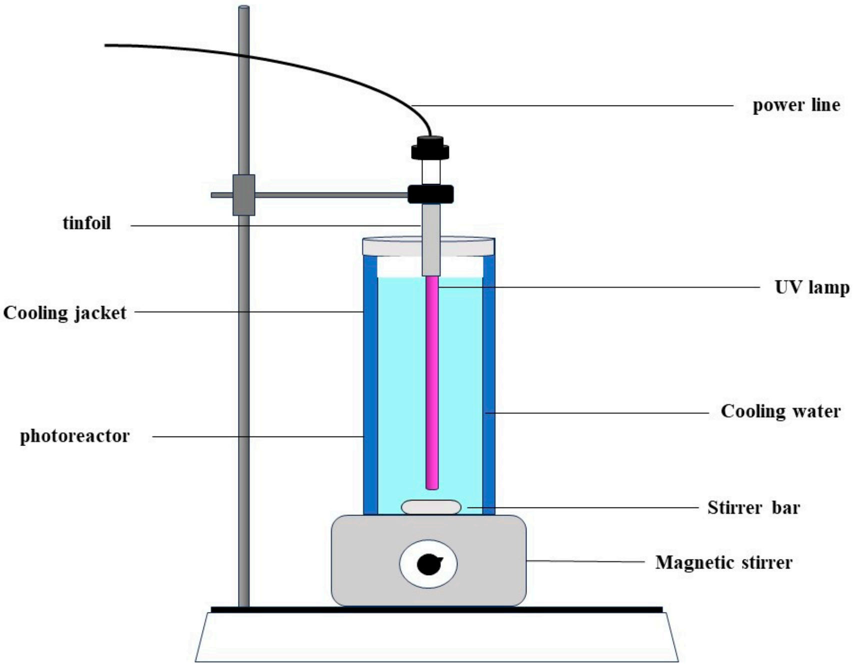

Figure S1 Photodegradation device

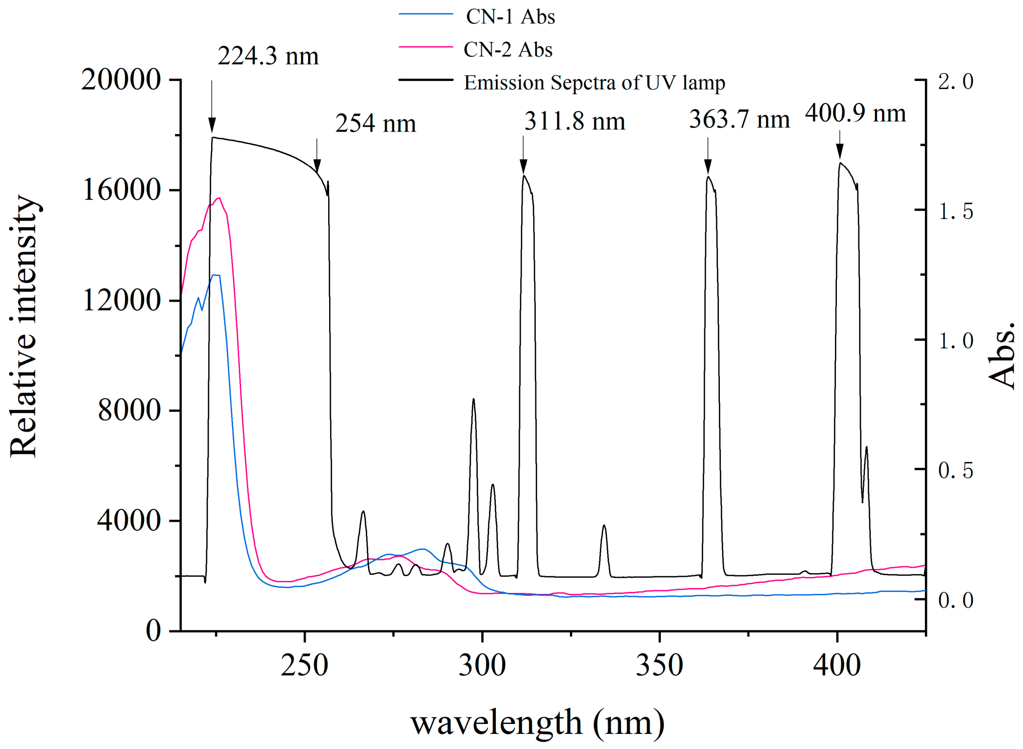

Figure S2 UV–C lamp emission spectrum, UV absorption spectra of CN–1 and CN–2

(Conditions: $[CN–1]_0 = 1$ mg L^{-1}, $[CN–2]_0 = 1$ mg L^{-1} in methanol solution, 1cm cuvette)

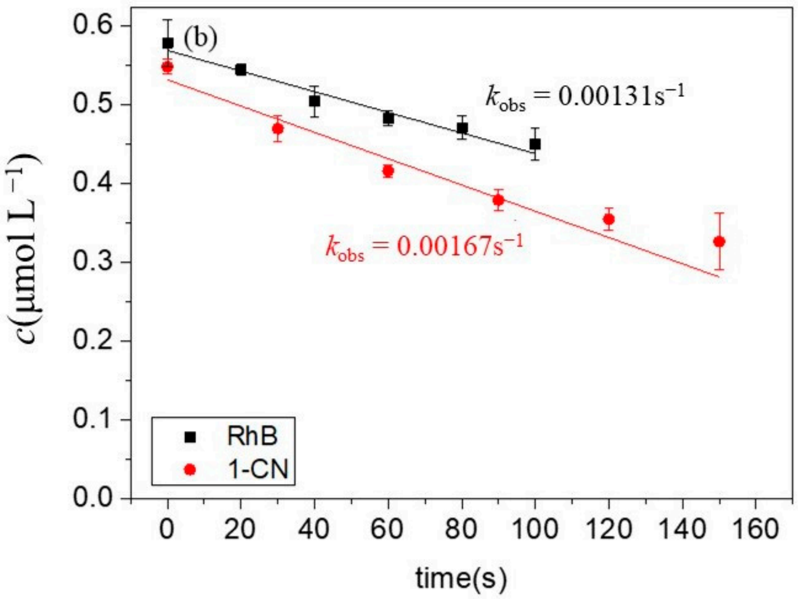
(b)
k_{obs} = 0.00131s^{-1}
k_{obs} = 0.00167s^{-1}
c(μmol L^{-1})
0.6
0.5
0.4
0.3
0.2
0.1
0.0
0
20
40
60
80
100
120
140
160
time(s)
RhB
1-CN

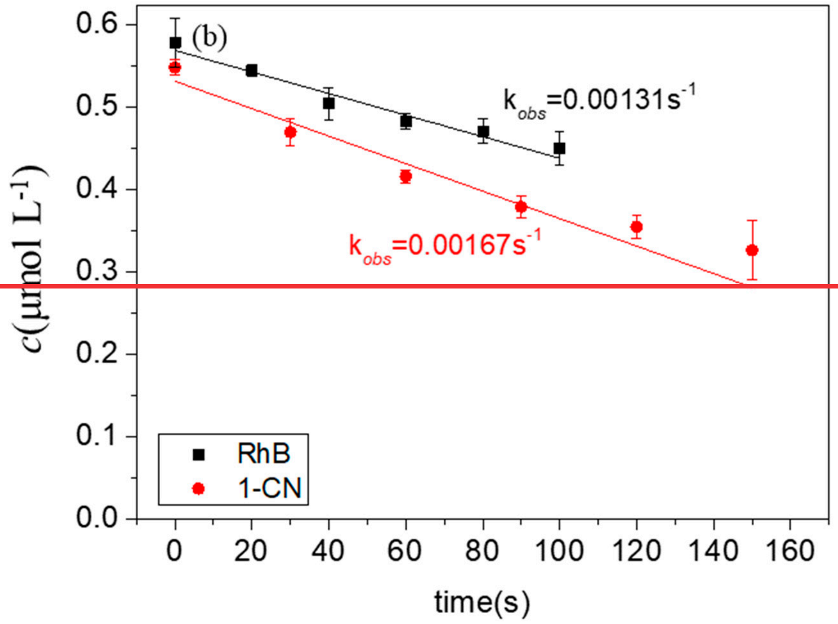
(b)
k_{obs}=0.00131s^{-1}
k_{obs}=0.00167s^{-1}
c(μmol L^{-1})
0.6
0.5
0.4
0.3
0.2
0.1
0.0
0
20
40
60
80
100
120
140
160
time(s)
RhB
1-CN

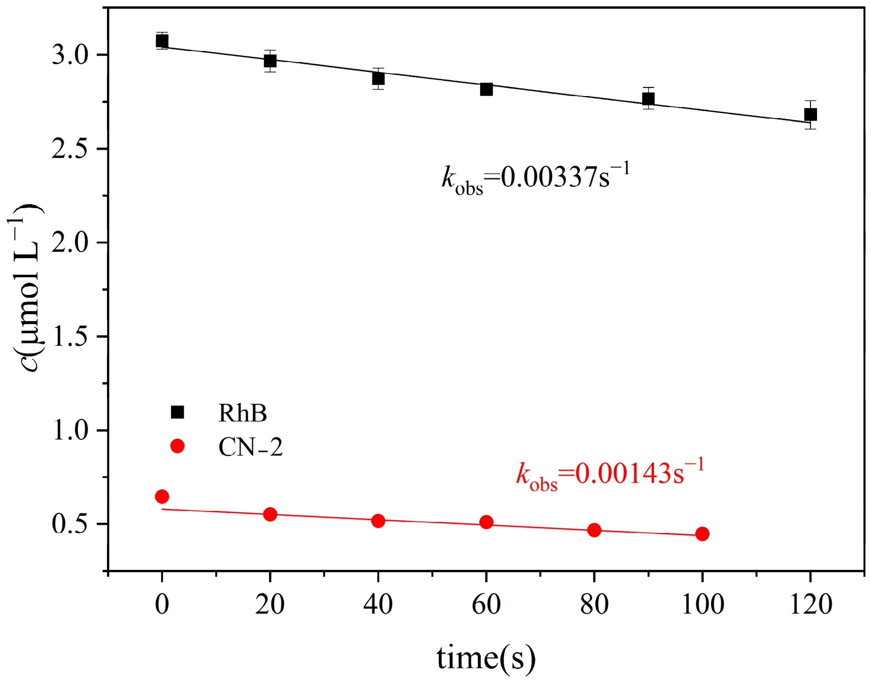

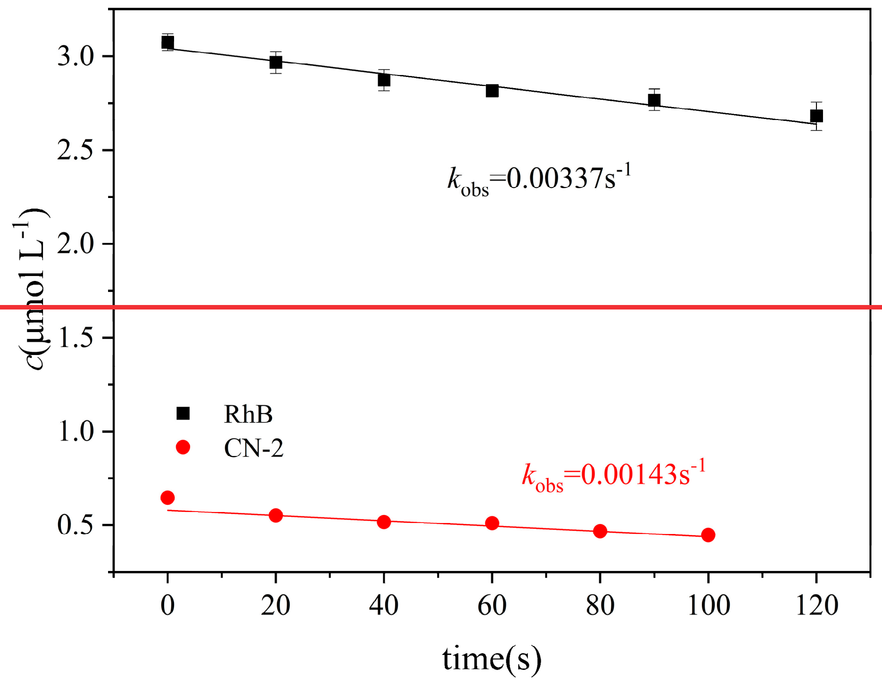

Figure S3 Concentration variation in a competitive reaction (**a**)CN–1 and RhB; (**b**)CN–2 and RhB

Conditions: $[CN\text{–}1]_0$ = 0.615μmol L^{-1}, $[RhB]_0$ = 0.552μmol L^{-1}; $[CN\text{–}2]_0$ = 0.615μmol L^{-1}, $[RhB]_0$ = 3.075μmol L^{-1};20°C

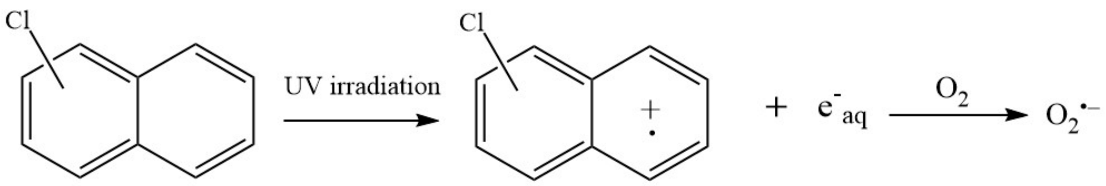

Figure **S4** Scheme for generating $O_2^{\bullet-}$ by monochlorinated naphthalene under UV irradiation

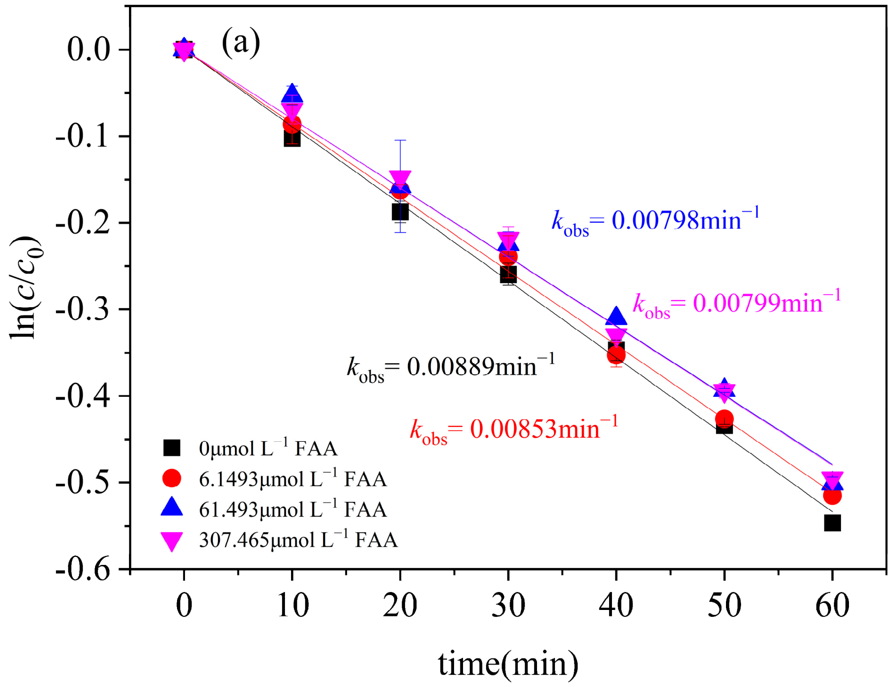

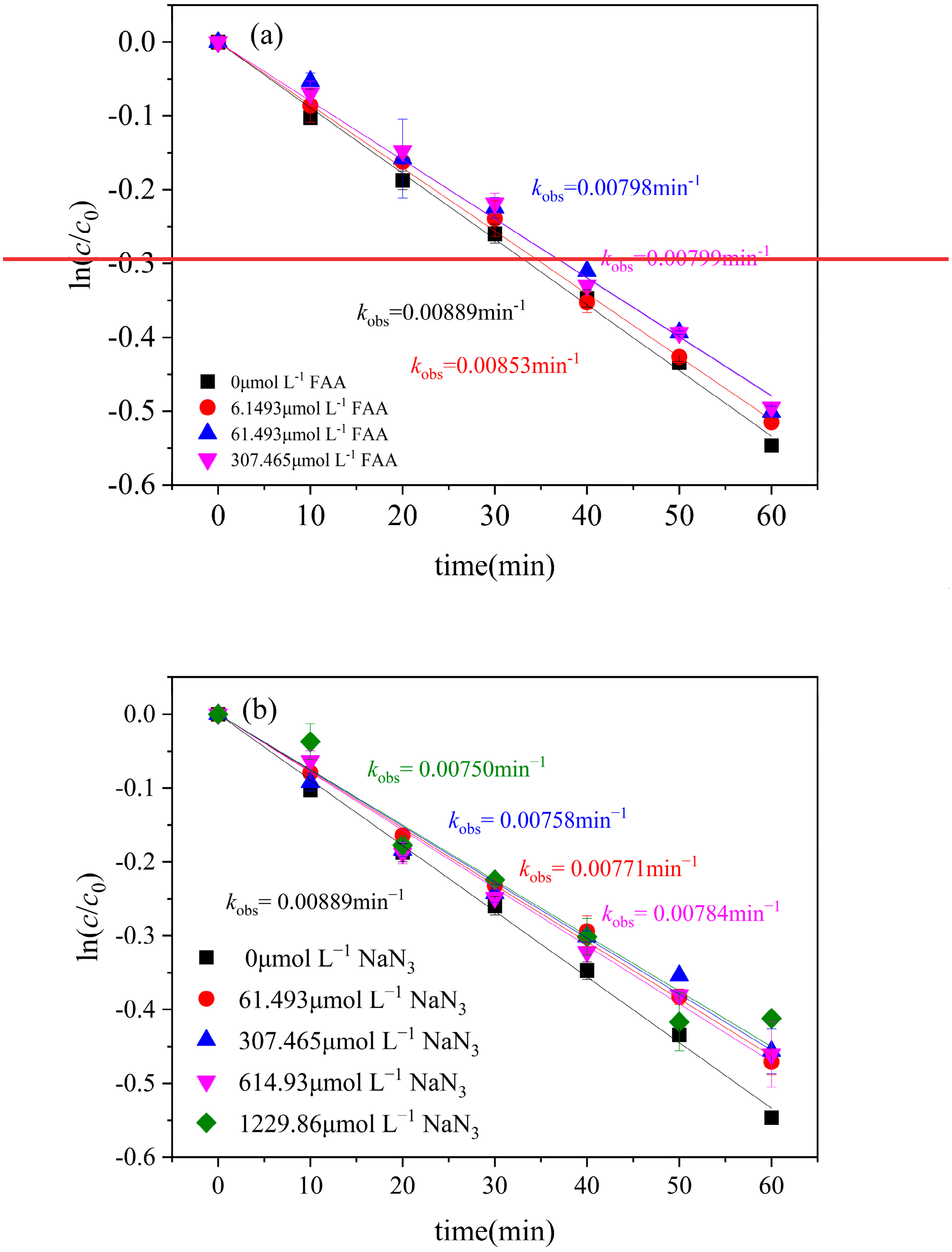
(a)
k_{obs}=0.00798min^{-1}
k_{obs}=0.00799min^{-1}
k_{obs}=0.00889min^{-1}
k_{obs}=0.00853min^{-1}
0μmol L^{-1} FAA
6.1493μmol L^{-1} FAA
61.493μmol L^{-1} FAA
307.465μmol L^{-1} FAA
ln(c/c_0)
time(min)
(b)
k_{obs}= 0.00750min^{-1}
k_{obs}= 0.00758min^{-1}
k_{obs}= 0.00771min^{-1}
k_{obs}= 0.00889min^{-1}
k_{obs}= 0.00784min^{-1}
0μmol L^{-1} NaN_3
61.493μmol L^{-1} NaN_3
307.465μmol L^{-1} NaN_3
614.93μmol L^{-1} NaN_3
1229.86μmol L^{-1} NaN_3
ln(c/c_0)
time(min)

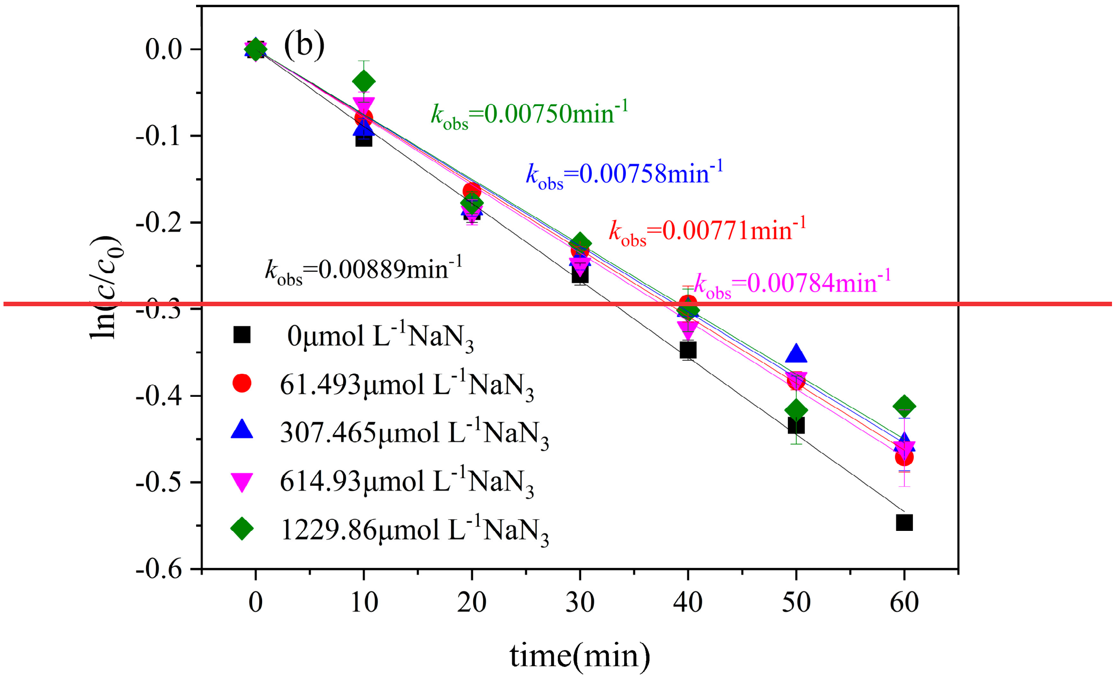

Figure S5 Effects of $^1O_2$ scavengers on photodegradation of CN–1 (**a**)FFA (**b**)NaN_3

Conditions: $[CN–1]_0 = 0.615$μmol L $^{-1}$, 20°C

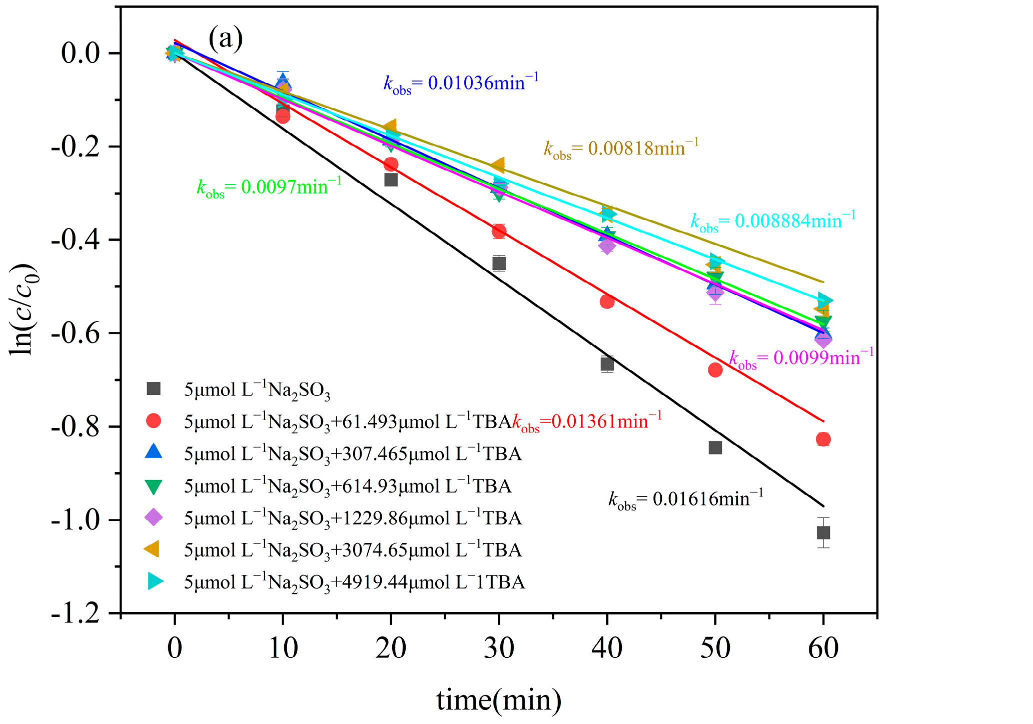

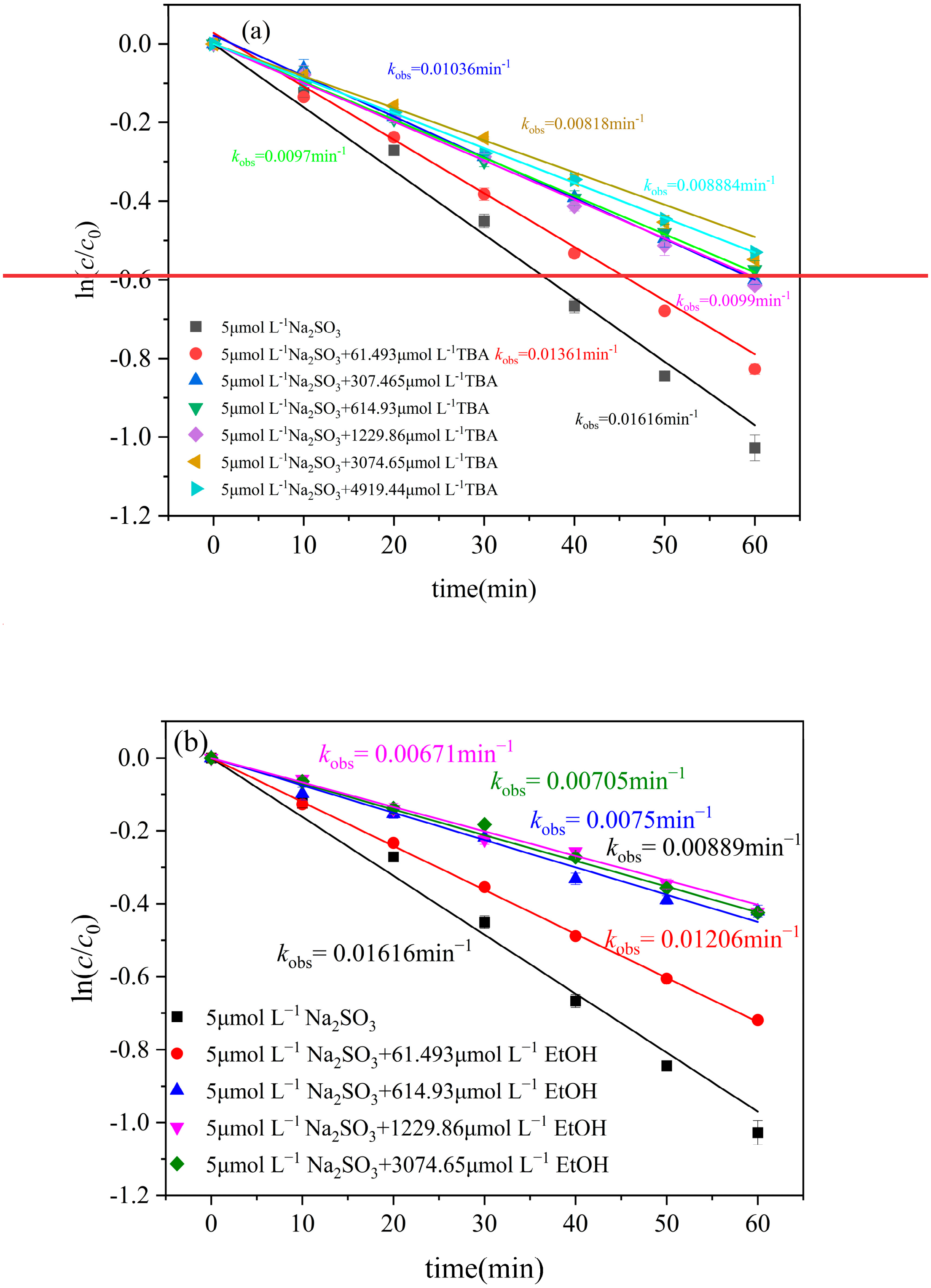
(a)
0.0
-0.2
-0.4
-0.6
-0.8
-1.0
-1.2
ln(c/c0)
0
10
20
30
40
50
60
time(min)
kobs=0.01036min-1
kobs=0.00818min-1
kobs=0.0097min-1
kobs=0.008884min-1
kobs=0.0099min-1
kobs=0.01361min-1
kobs=0.01616min-1
5μmol L-1Na2SO3
5μmol L-1Na2SO3+61.493μmol L-1TBA
5μmol L-1Na2SO3+307.465μmol L-1TBA
5μmol L-1Na2SO3+614.93μmol L-1TBA
5μmol L-1Na2SO3+1229.86μmol L-1TBA
5μmol L-1Na2SO3+3074.65μmol L-1TBA
5μmol L-1Na2SO3+4919.44μmol L-1TBA
(b)
0.0
-0.2
-0.4
-0.6
-0.8
-1.0
-1.2
ln(c/c0)
0
10
20
30
40
50
60
time(min)
kobs= 0.00671min−1
kobs= 0.00705min−1
kobs= 0.0075min−1
kobs= 0.00889min−1
kobs= 0.01206min−1
kobs= 0.01616min−1
5μmol L−1 Na2SO3
5μmol L−1 Na2SO3+61.493μmol L−1 EtOH
5μmol L−1 Na2SO3+614.93μmol L−1 EtOH
5μmol L−1 Na2SO3+1229.86μmol L−1 EtOH
5μmol L−1 Na2SO3+3074.65μmol L−1 EtOH

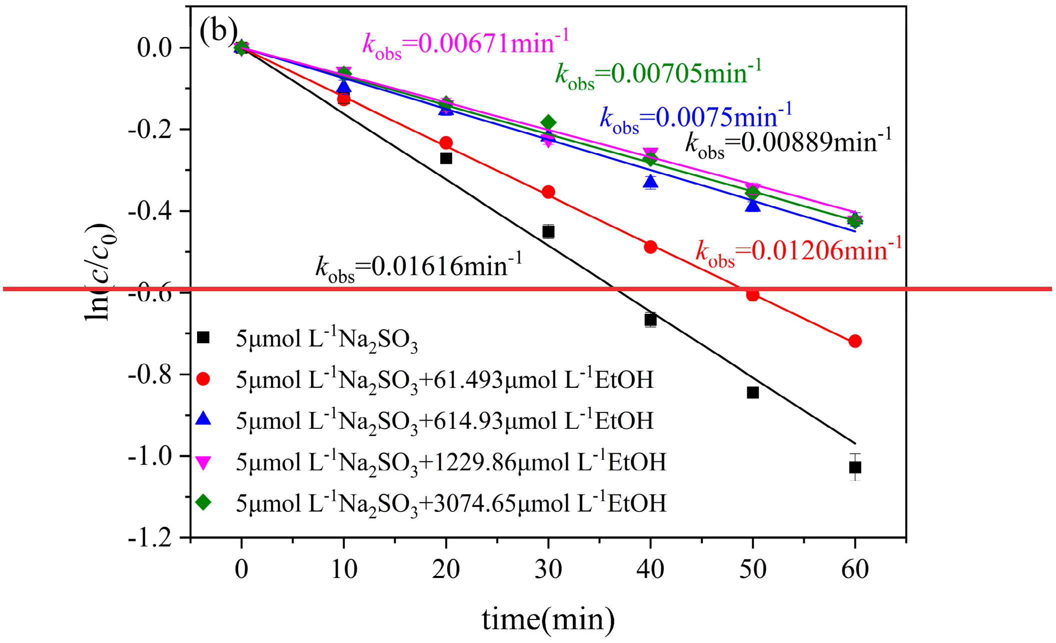

Figure S6 Effects of scavengers on the photodegradation of CN–1 with Na_2SO_3(**a**) TBA; (**b**) EtOH

Conditions: $[CN–1]_0$ = 0.615μmol L^{-1}, 20℃

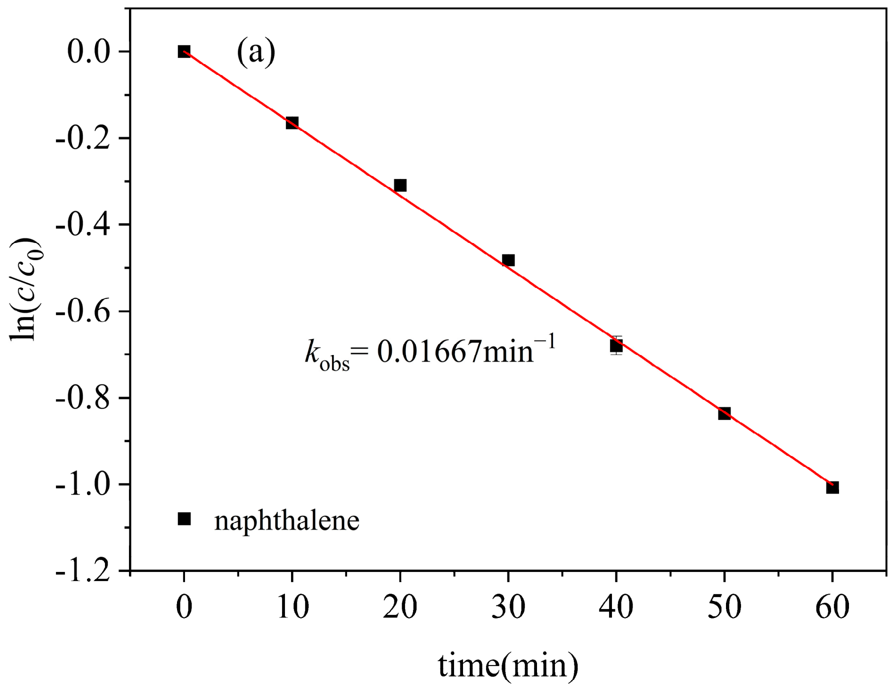

(a)
k_{obs}= 0.01667min^{-1}
naphthalene
ln(c/c_0)
time(min)

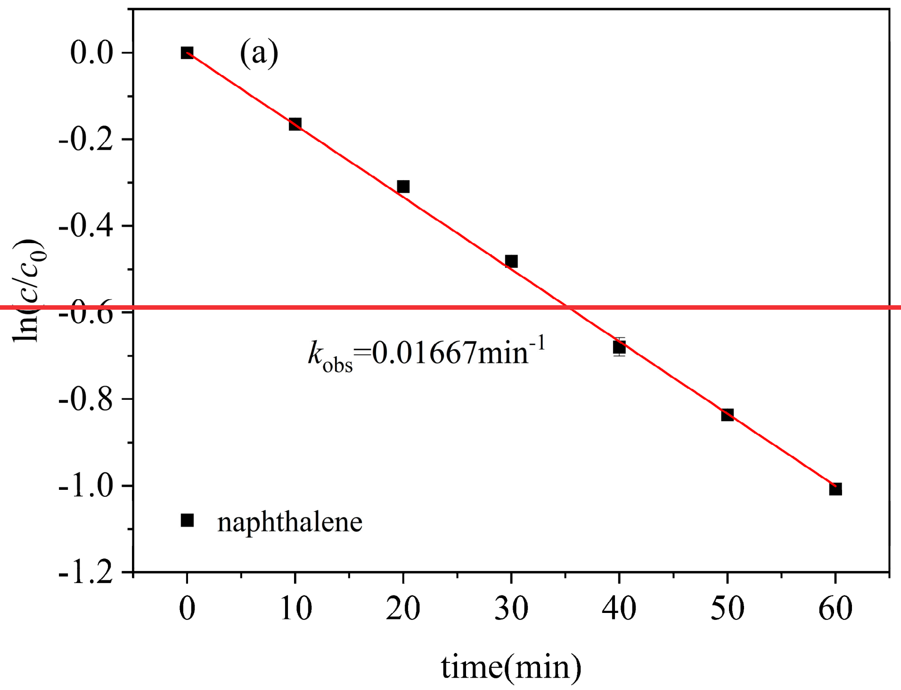

(a)
k_{obs}=0.01667min^{-1}
naphthalene
ln(c/c_0)
time(min)

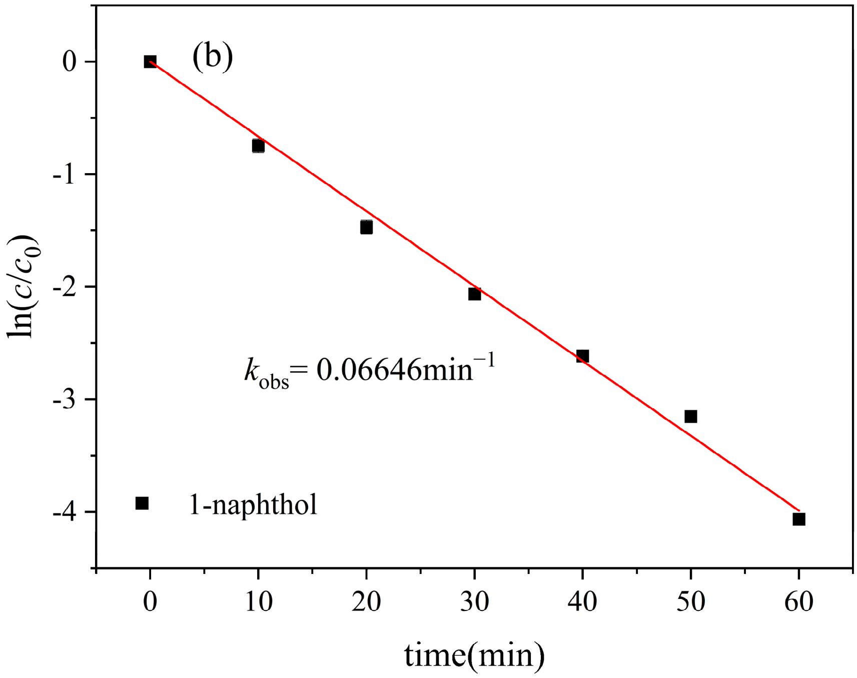

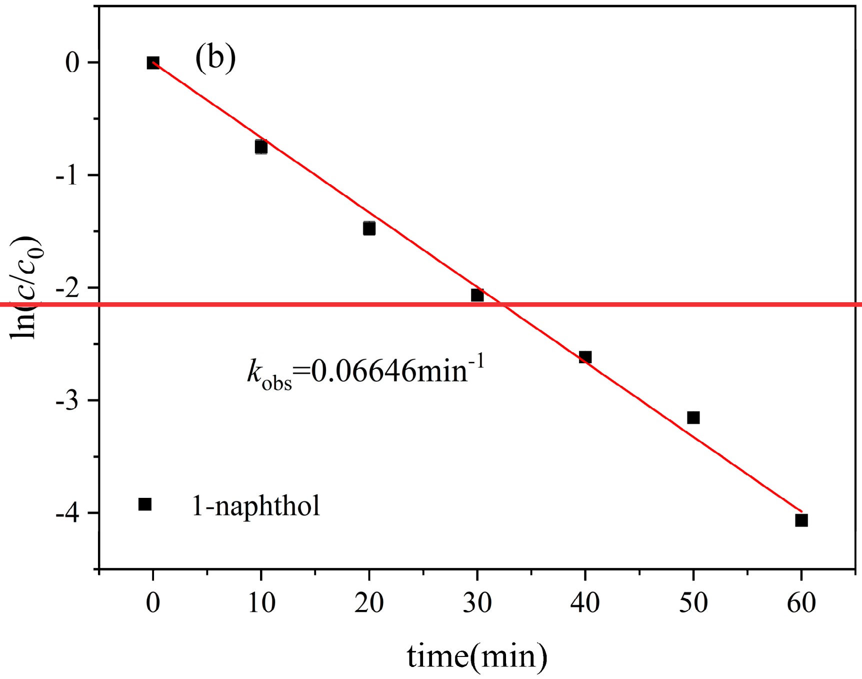

Figure S7 photodegradation experiment (**a**) naphthalene (**b**) 1−naphthol

Conditions: [Naphthalene]$_0$ = 0.615μmol L^{-1} [1–Naphthol]$_0$ = 0.615μmol L^{-1}, 20°C

Text S1. Specific use of reagents

In this study, CN–1, (98% purity), and CN–2, (98% purity) were used as the substrate. 1–naphthol (98% purity), and naphthalene (98% purity) were used as product analysis. The methanol was used as the mobile phase and solvent (The mobile phase was methanol and water (90:100, v/v). And methanol was used as a solvent for the UV absorption spectra of CN–1 and CN–2.). The IPA and H_2O_2 (30%, v/v) were used to explore the role of $^{\bullet}OH$. 2 mol L^{-1} of perchloric acid, and 2 mol L^{-1} of sodium hydroxide was used to adjust the pH of the solution. The NaCl, $NaSO_4$, Na_2SO_3, Na_2CO_3, and $NaNO_3$ were used to investigate the effect of anions on CN–1 photodegradation. Rhodamine B(RhB) was used as the competitive chemical to react with $^{\bullet}OH$. The competitive reaction is the use of CN–1, CN–2, and RhB in the Fenton system (hydrogen peroxide (30%, *v/v*) and ferrous sulfate heptahydrate) to compete for •OH and determine the value of $k_{(CN–1, ^{\bullet}OH)}$ and $k_{(CN–2, ^{\bullet}OH)}$. The $^{\bullet}OH$ was quenched by adding TBA as scavengers, and the $SO_4^{\bullet-}$ and $^{\bullet}OH$ were quenched by adding EtOH as scavengers. $^1O_2$ was quenched by adding FAA and NaN_3 as scavengers during the photodegradation of CN–1. Acetonitrile was used as a reaction solution. (Reactions were initiated by adding 0.615μmol L^{-1} CN–1/CN–2 into solutions containing acetonitrile-water (1‰, *v/v*)). In experiments, all solutions were prepared with ultrapure water (⩾ 18.2 MΩ cm).
